# Multi-omic Profiling Reveals that Intra-abdominal-Hypertension-Induced Intestinal Damage Can Be Prevented by Microbiome and Metabolic Modulations with 5-Hydroxyindoleacetic Acid as a Diagnostic Marker

**DOI:** 10.1128/msystems.01204-21

**Published:** 2022-05-16

**Authors:** Fang Li, Liuyiqi Jiang, Shuming Pan, Shaowei Jiang, Yiwen Fan, Chao Jiang, Chengjin Gao, Yuxin Leng

**Affiliations:** a Department of Intensive Care Unit, Peking University Third Hospital, Beijing, China; b Department of Hematology, Peking University Third Hospital, Beijing, China; c Life Sciences Institute, Zhejiang University, Hangzhou, China; d Department of Emergency, Xinhua Hospital Affiliated to Shanghai Jiao Tong University School of Medicine, Shanghai, China; Duke University

**Keywords:** intra-abdominal hypertension, sepsis, biomarker, multi-omics, microbiome, tryptophan metabolism, 5-hydroxyindoleacetic acid (5-HIAA)

## Abstract

Emerging evidence shows that modulation of the microbiome can suppress intra-abdominal hypertension (IAH)-induced intestinal barrier damage through the regulation of amino acid (AA) biosynthesis. Here, we investigated the protective effects of orally gavaged Lactobacillus acidophilus L-92 (L92) and a mixture of AA in rats with induced IAH. The results showed that both L92 and AA pretreatments effectively mitigated IAH-induced intestinal damage. Interestingly, L92 but not AA prevented metagenomic changes induced by IAH. Bacteroides fragilis, Bacteroides eggerthii, Bacteroides ovatus, Faecalibacterium prausnitzii, *Prevotella*, and extensively altered functional pathways were associated with L92-mediated host protection. Metabolomic profiling revealed that tryptophan metabolism was involved in both L92- and AA-mediated gut protection. The tryptophan metabolite 5-hydroxyindoleacetic acid (5-HIAA) is a sensitive biomarker for IAH in rats and patients with either gut-derived sepsis (*n* = 41) or all-source sepsis (*n* = 293). In conclusion, we show that microbiome and metabolic modulations can effectively prevent IAH-induced intestinal damage and that 5-HIAA is a potential metabolic marker for IAH and sepsis.

**IMPORTANCE** Gut protection through modulation of the microbiome for critically ill patients has been gaining much attention recently. Intra-abdominal hypertension (IAH) is a prevailing clinical feature of acute gastrointestinal injuries in critically ill patients, characterized by nonspecific intestinal barrier damage. Prolonged IAH can induce or aggravate the development of sepsis and multiorgan dysfunctions. Therefore, the prevention of IAH-induced damage in rats through microbiome and metabolic interventions by commercially available L92 and AA treatments and the identification of 5-HIAA as an important marker for IAH/sepsis have important clinical implications for the treatment and early diagnosis of critically ill patients.

## INTRODUCTION

Intra-abdominal hypertension (IAH) (intra-abdominal pressure [IAP] above 12 mm Hg in adults and above 10 mm Hg in children) is a common complication in the intensive care unit (ICU) that directly contributes to early organ dysfunction and is frequently associated with mortality in critically ill patients (1). Previous studies have demonstrated that the pathophysiological mechanisms might be related to disturbed intestinal barrier functions induced by IAH, which is considered to be the origin of gut-derived sepsis (2, 3). Thus, prevention of IAH-induced intestinal damage could provide an effective strategy for inhibiting the deterioration of IAH and even suppressing the development of gut-derived sepsis.

The gut microbiota, a key component of the intestinal biological barrier, has physiological functions associated with nutrition metabolism, immune responses, and defense of the host (4). The gut microbiota can sense tiny changes in the intestinal microenvironment that may lead to intestinal infection when the microecological balance is disturbed by an external or internal stimulus (5). Microbiome-based interventions, whether by reshaping the composition of the microbial community or by modifying the function of the microbiota, can inhibit further aggravation of critical illness (6–9). Nevertheless, whether prophylactic modifications of the microbiome will protect the gut or even reduce the probability of gut-derived sepsis needs to be clarified. This is especially useful for microbiome-related clinical translation research because microbiome-based predisease prevention will greatly reduce the risks associated with fecal transplant after the onset of disease, which can frequently compromise the immune system. In our previous IAH study, we found that IAH-induced intestinal damage can be alleviated by pretreatment with Lactobacillus acidophilus, potentially through the activation of amino acid (AA) biosynthesis (2, 10), but the sample size was limited. Therefore, prevention of IAH-induced gut damage may be achieved by regulating the microbiome through simple supplementation with probiotics or amino acids.

To test these assumptions, groups of Sprague-Dawley (SD) rats (*n* = 20 in each group) were pretreated with L92 (single-species probiotic [Lactobacillus acidophilus L-92]) or AA (commercialized enteral nutrition with mixed amino acids; Elental) for 7 days (oral gavage, twice daily) and subjected to nitrogen pneumoperitoneum for 90 min to induce IAH. First, we confirmed the protective effects of L92 and AA based on pathological changes in intestinal histology. Next, we explored the involvement of the microbiota and metabolome with multi-omics approaches, revealed the features of taxonomy and metabolomic pathways associated with host protection, and identified 5-hydroxyindoleacetic acid (5-HIAA) as a key metabolic marker. Finally, we demonstrated that 5-HIAA can be used as a biomarker for the diagnosis of IAH and even sepsis in patients.

## RESULTS

### L92 and AA pretreatments effectively mitigated IAH-induced intestinal damage.

**FIG 1:**
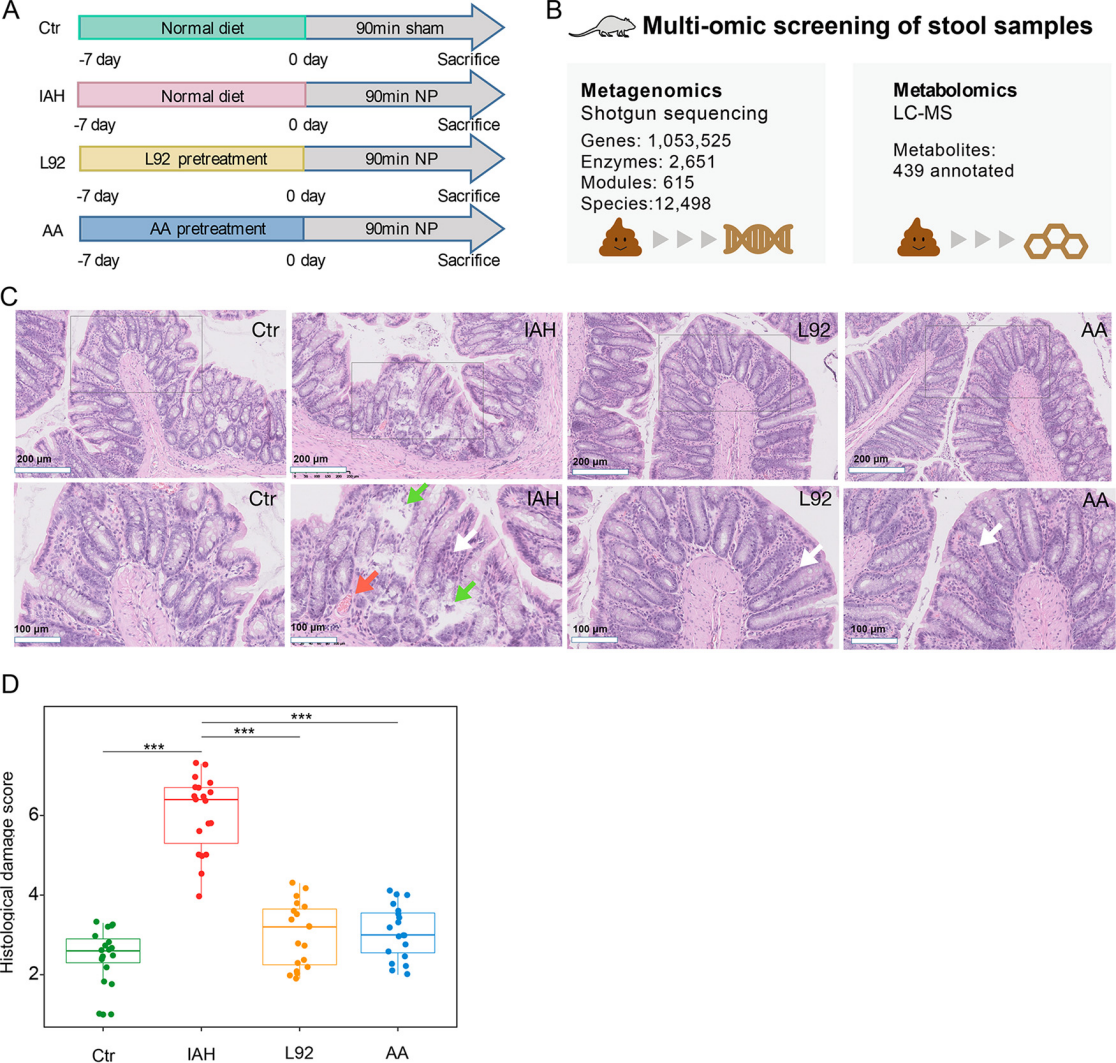
L92 and AA pretreatments mitigated IAH-induced intestinal barrier damage. (A) Male Sprague-Dawley rats (8 to 10 weeks old; *n* = 20 in each group) were pretreated with L92 or AA by intragastric gavage twice daily, starting 1 week before inducing acute IAH (exposure to 12 mm Hg nitrogen pneumoperitoneum [NP] for 90 min). (B) Schematic of the study design. (C) Histological changes. L92 and AA pretreatments mitigated IAH-induced inflammatory infiltration, mucosal congestion, and epithelial integrity. Gray boxes indicate the magnified images below. The red arrow indicates mucosal congestion. Green arrows indicate digested lamina propria. White arrows indicate inflammatory infiltration. (D) Comparison of histological damage scores.

To assess the effect of L92/AA in preventing IAH-induced intestinal damage, the nitrogen pneumoperitoneum rat model was adopted to induce IAH (11) (Fig. 1A). Pathological changes in histology and physiological indices associated with systematic or colonic inflammation and oxidative injuries were analyzed (12, 13). Metagenomic and metabolomic data were integrated to investigate the role of microbiome and metabolome modulations in L92- and AA-mediated gut protection.

In total, 76 stool samples (*n* = 20 in the control [Ctr] group, *n* = 20 in the IAH group, *n* = 18 in the L92 group, and *n* = 18 in the AA group) were subjected to whole-metagenome sequencing, and 1,053,525 genes, 2,651 enzymes, and 615 modules were identified (Fig. 1B). Seventy-one stool samples (*n* = 17 in the control group, *n* = 17 in the IAH group, *n* = 19 in the L92 group, and *n* = 18 in the AA group) were subjected to metabolomics profiling by untargeted liquid chromatography-mass spectrometry (LC-MS), and 439 metabolites were annotated. Induced IAH resulted in significant pathological damage compared to the control group. As shown in Fig. 1C, inflammatory infiltration, mucosal congestion, and disruption of mucosal integrity under IAH were effectively mitigated by both L92 and AA pretreatments, resulting in a significant decrease in the histological damage score (Fig. 1D). Seven-day pretreatment with L92 prevented weight loss after IAH (see Fig. S1A in the supplemental material). The IAH-induced increase in interleukin-1β (IL-1β) was significantly alleviated by AA supplementation (Fig. S1B). For systematic diamine oxidase (DAO), lactic acid (LA), and colonic glutathione (GSH)/malondialdehyde (MDA), significant differences between groups were found (Fig. S1C to G). These results demonstrated that L92 and AA pretreatments effectively mitigated IAH-induced damage in rats.

10.1128/msystems.01204-21.1 FIG S1 Measured indices reflecting systematic/colonic inflammation and oxidative injuries after IAH. Shown are measured weight (A), DAO (B), LA (C), IL-1β (D), GSH (E), MDA (F), and GSH/MDA ratios (G) in the four groups involved. DAO and LA were measured to assess intestinal permeability. IL-1β is an immunomodulatory marker. GSH, MDA, and GSH/MDA reflect oxidative balance. Data are shown as means ± SEM. Download FIG S1, TIF file, 0.5 MB. Copyright © 2022 Li et al. 2022 Li et al. https://creativecommons.org/licenses/by/4.0/ This content is distributed under the terms of the Creative Commons Attribution 4.0 International license.

### L92 pretreatment prevents IAH-induced microbiota alterations.

**FIG 2:**
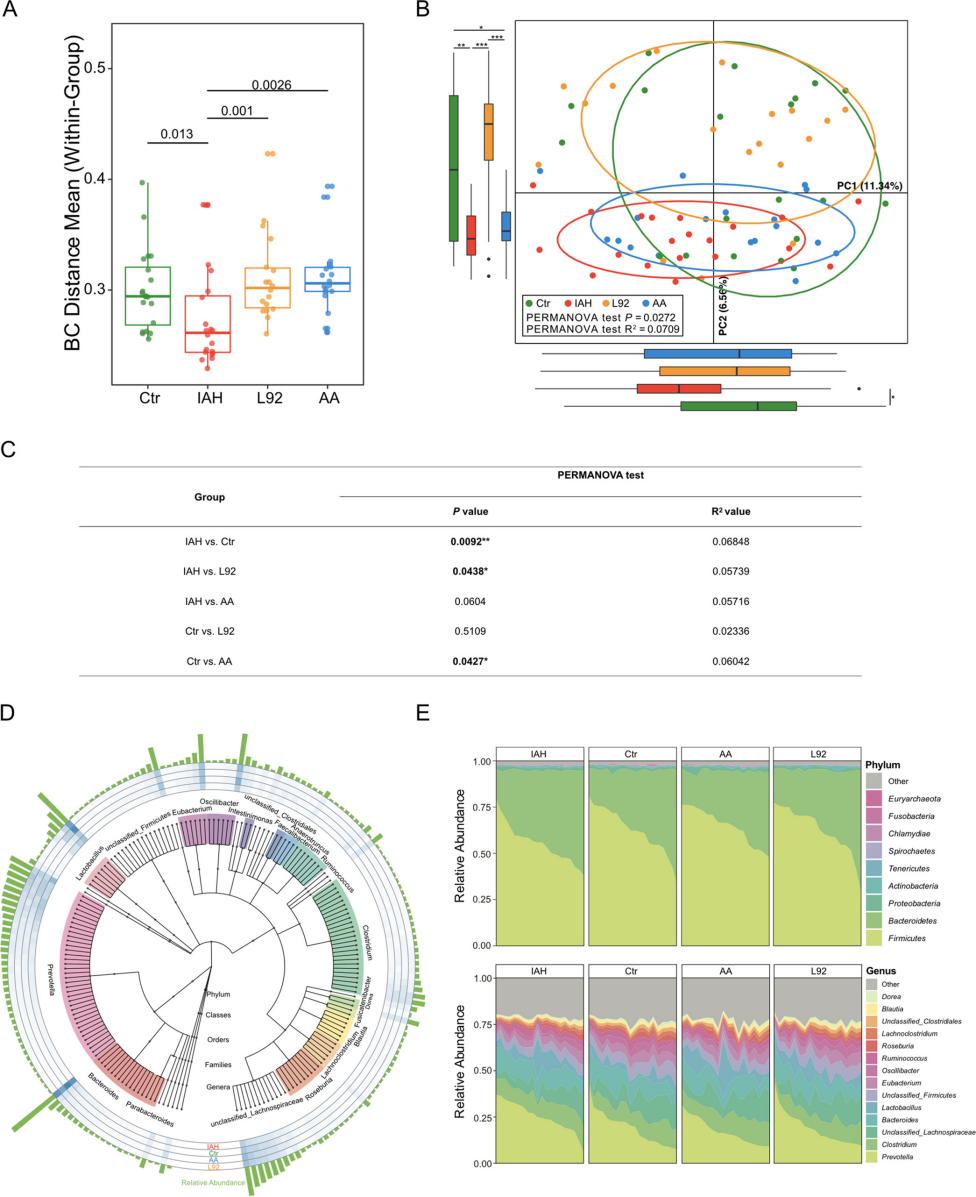
Influence of L92 and AA on IAH-induced microbiota alterations in rats. (A) Box plots of community variability within each group based on Bray-Curtis (BC) distance. (B) Species-based principal-component analysis (PCA) of the IAH, Ctr, L92, and AA groups. Box plots show the overall distribution of PC1 and PC2 scores. (C) Adonis analysis of species abundance profiles using Euclidean distance matrices. The significance of a comparison of two groups was evaluated with 999 permutations (*, *P < *0.05; **, *P < *0.01). (D) Phylogenetic tree constructed from the metagenomic profile. Colored blocks of the inner circle indicate genera. Bar plots of the outermost circle show the mean abundances of all samples. The heatmap circles show the relative abundance of each species in the IAH, Ctr, AA, and L92 groups. (E) Area plots of microbial composition at the phylum level (top) and the genus level (bottom) in the four groups.

To explore the potential mechanisms of the protective effects of L92 and AA pretreatments, we first examined the gut microbiota. The intragroup variation (Bray-Curtis distance) of the gut microbiota in the IAH group was significantly lower than that in the control group, but the L92 and AA groups showed no significant differences compared to the control group (Fig. 2A). Principal-component analysis (PCA) demonstrated a significant difference in the overall community composition of the microbiome between the control and IAH groups (Fig. 2B and C) (*P < *0.05 by an Adonis test with Euclidean distance). The microbial profiles of the L92 group were more similar to those of the control group, while those of the AA group were more similar to those of the IAH group.

Relative abundance profiles show that *Firmicutes*, *Bacteroidetes*, *Proteobacteria*, *Actinobacteria*, and *Tenericutes* were the major phyla; *Prevotella*, *Clostridium*, unclassified *Lachnospiraceae*, *Bacteroides*, and *Lactobacillus* were the most abundant genera (Fig. 2D and E). A total of 334 genera were differentially abundant between the IAH group and the other three groups (adjusted *P* value of <0.05 by Tukey’s honestly significant difference [HSD] test). As expected, the L92 group was more similar to the control group, while the AA group shared only 14 genera with the control group (Fig. S2A and B). In addition, we constructed a correlational network based on the relative abundance profiles of differentially abundant genera shared by the L92 and control groups (Fig. S2C). In the network, species were positively or negatively correlated with each other, suggesting diverse ecological relationships. These findings indicated that L92 but not AA pretreatment could prevent IAH-induced microbiota changes.

10.1128/msystems.01204-21.2 FIG S2 Differences in the gut microbiota at the genus level. (A) Venn diagrams showing the shared differentially abundant genera among the control, L92, and AA groups compared to the IAH group. (B) Box plots showing the differentially abundant genera shared by the L92 and control groups. (C) Cooccurrence network of the differentially abundant genera between the IAH group and the L92 or control group. Red edges indicate positive correlations; blue edges indicate negative correlations. Spearman analysis was used to calculate the correlations. The *P* values of the correlation results were adjusted by the FDR method. Statistical significance was set as an FDR-adjusted *P* value of <0.05. The network shows only the edges with a Spearman rank correlation coefficient of greater than 0.6 or less than −0.6. Download FIG S2, TIF file, 1.7 MB. Copyright © 2022 Li et al. 2022 Li et al. https://creativecommons.org/licenses/by/4.0/ This content is distributed under the terms of the Creative Commons Attribution 4.0 International license.

### L92 pretreatment beneficially modulates the gut microbiota.

**FIG 3:**
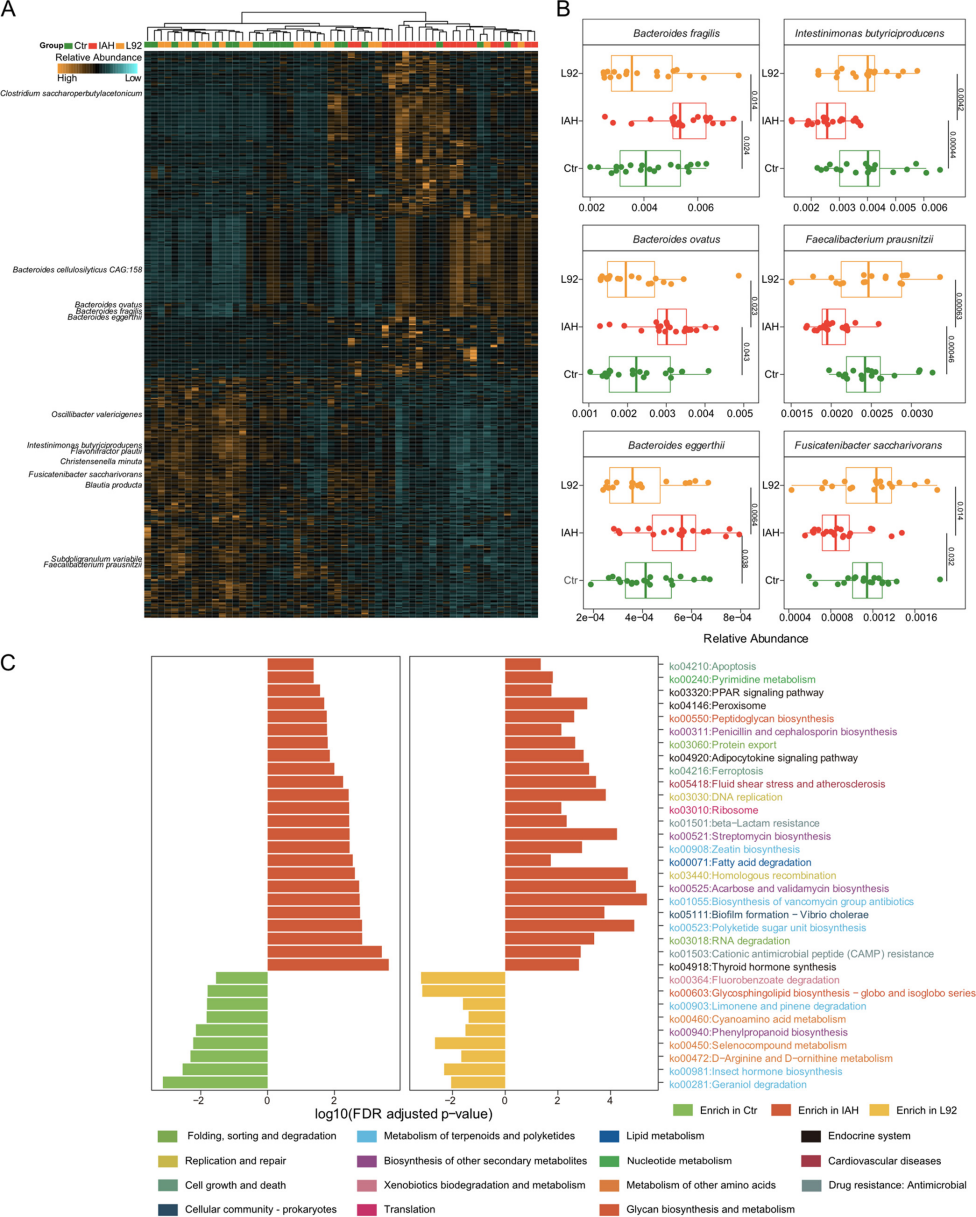
IAH-mediated microbial and functional changes can be prevented by L92 pretreatment. (A) Heatmap depicting the differentially abundant species among the IAH, L92, and control groups. Columns are ordered by hierarchical clustering. Each lattice represents the relative abundance of the microbe in a sample; yellow indicates a high abundance, and blue indicates a low abundance. (B) Representative differentially abundant species among the IAH, L92, and control groups. (C) Bar plots showing differentially abundant KEGG orthology (KO) pathways in the L92 and control groups compared to the IAH group. The colors of KO pathways represent KEGG pathway level B.

In order to better understand the influences of L92 pretreatment on the gut microbiota, we examined the differentially abundant species shared by the L92 and control groups compared to the IAH group. At the species level, the IAH group showed a remarkably distinct pattern compared to the L92 and control groups (Fig. 3A). In total, 277 species were present at a substantially higher abundance in the IAH group, such as *Bacteroides* spp. and Clostridium saccharoperbutylacetonicum (Fig. 3B and Fig. S3). Interestingly, several species of the *Prevotella* genus were significantly increased in the IAH group (Fig. S4A), although there were no significant changes at the *Prevotella* genus level (Fig. S4B). In contrast, 205 species significantly declined in abundance in the IAH group but were rescued by L92 pretreatment, including multiple species of *Clostridia*, Bacteroides fragilis, and Faecalibacterium prausnitzii (Fig. 3B and Fig. S3), suggesting their protective roles in IAH rats. In addition, we found that L92 pretreatment increased the abundance of Akkermansia muciniphila (Fig. S4C). A. muciniphila is known to produce specific short-chain fatty acids (SCFAs) that are linked to improved gut barrier function (14). In addition, we investigated the putative correlations between the abundances of differentially abundant species and host factors. Multiple species of the *Clostridium* genus showed strong positive associations with the histological damage score (Fig. S4D). These results demonstrated that L92 pretreatment modulated the gut microbiota in a beneficial way.

10.1128/msystems.01204-21.3 FIG S3 Examples of differentially abundant species among the IAH, L92, and Ctr groups. Download FIG S3, TIF file, 0.8 MB. Copyright © 2022 Li et al. 2022 Li et al. https://creativecommons.org/licenses/by/4.0/ This content is distributed under the terms of the Creative Commons Attribution 4.0 International license.

10.1128/msystems.01204-21.4 FIG S4 Alterations in the microbiota associated with L92 pretreatment. (A) Bar plots showing the differentially abundant species of the *Prevotella* genus (*, *P < *0.05; **, *P < *0.01; ***, *P < *0.001). (B and C) Box plots showing the relative abundances of *Prevotella* (B) and Akkermansia muciniphila (C). (D) Heatmap showing correlations between differentially abundant species and measured host factors. Positive and negative correlations are shown in red and blue in the heatmap, respectively (*, *P < *0.05; **, *P < *0.01; ***, *P < *0.001 [all *P* values are adjusted by FDR correction]). Download FIG S4, TIF file, 1.0 MB. Copyright © 2022 Li et al. 2022 Li et al. https://creativecommons.org/licenses/by/4.0/ This content is distributed under the terms of the Creative Commons Attribution 4.0 International license.

### L92 pretreatment alters microbial functional characteristics.

Similar to the community analysis, the overall microbial functional profiles (KEGG) of the L92 group were close to those of the control, while those of the AA group were not, based on permutational multivariate analysis of variance (PERMANOVA) (Adonis test with Euclidean distance) and PCA results (Fig. S5A and B). To examine the functional consequences of microbial community changes, we identified KEGG pathways that differed significantly between the IAH and the control/L92 groups. Twenty-four pathways were significantly elevated in the IAH group relative to the L92/control groups; 9 pathways were significantly elevated in both the L92 and control groups (Fig. S5C). Among the KEGG pathways identified, most were involved in the metabolism of terpenoids and polyketides, the biosynthesis of other secondary metabolites, and the metabolism of other amino acids and the endocrine system. In the category of metabolism of other amino acids, selenocompound metabolism was particularly enriched in both the L92 and control groups (Fig. 3C). On the contrary, pathways involved in apoptosis, pyrimidine metabolism, the peroxisome proliferator-activated receptor (PPAR) signaling pathway, protein export, DNA replication, and cationic antimicrobial peptide resistance were enriched in the IAH group, indicating that the microbiota needed to fight against host immune responses. We also observed pathways enriched in the IAH group related to host factors such as the histological damage score and DAO (Fig. S5D).

10.1128/msystems.01204-21.5 FIG S5 Functional changes in the fecal microbiota induced by L92/AA and the potential importance of Trp metabolism defined by untargeted metabolomics. (A to D) Metagenomic analyses. (A) KO-based PCA of the IAH, control, L92, and AA groups. Box plots show the overall distribution of the PC1 and PC2 scores of the samples (*, *P < *0.05). (B) The significance of each pairwise comparison was evaluated by performing 999 permutations (*, *P < *0.05; **, *P < *0.01). (C) The heatmap and hierarchical clustering of KO pathways changed in the metagenome of the L92 and control groups compared to the IAH group. (D) Heatmap showing correlations between differentially abundant KO pathways and host factors. Positive and negative correlations are shown in red and blue in the heatmap, respectively (*, *P < *0.05; **, *P < *0.01; ***, *P < *0.001 [all *P* values are adjusted by FDR correction]). (E to G) Untargeted metabolomic analyses. (E) Significantly changed metabolites in the IAH group that were reversed by L92 pretreatment. (F) Metabolites significantly changed in the IAH group and reversed by AA pretreatment. (G) List of metagenome functional enzymes and pathways (level 3) correlated with the abundance of 2-formaminobenzoylacetate and 5-HIAA. Ko00380, tryptophan pathway; Ko04726, serotonergic pathway. Download FIG S5, TIF file, 1.5 MB. Copyright © 2022 Li et al. 2022 Li et al. https://creativecommons.org/licenses/by/4.0/ This content is distributed under the terms of the Creative Commons Attribution 4.0 International license.

### Metabolomic analysis revealed the Trp metabolite 5-HIAA as a key marker for IAH.

**FIG 4:**
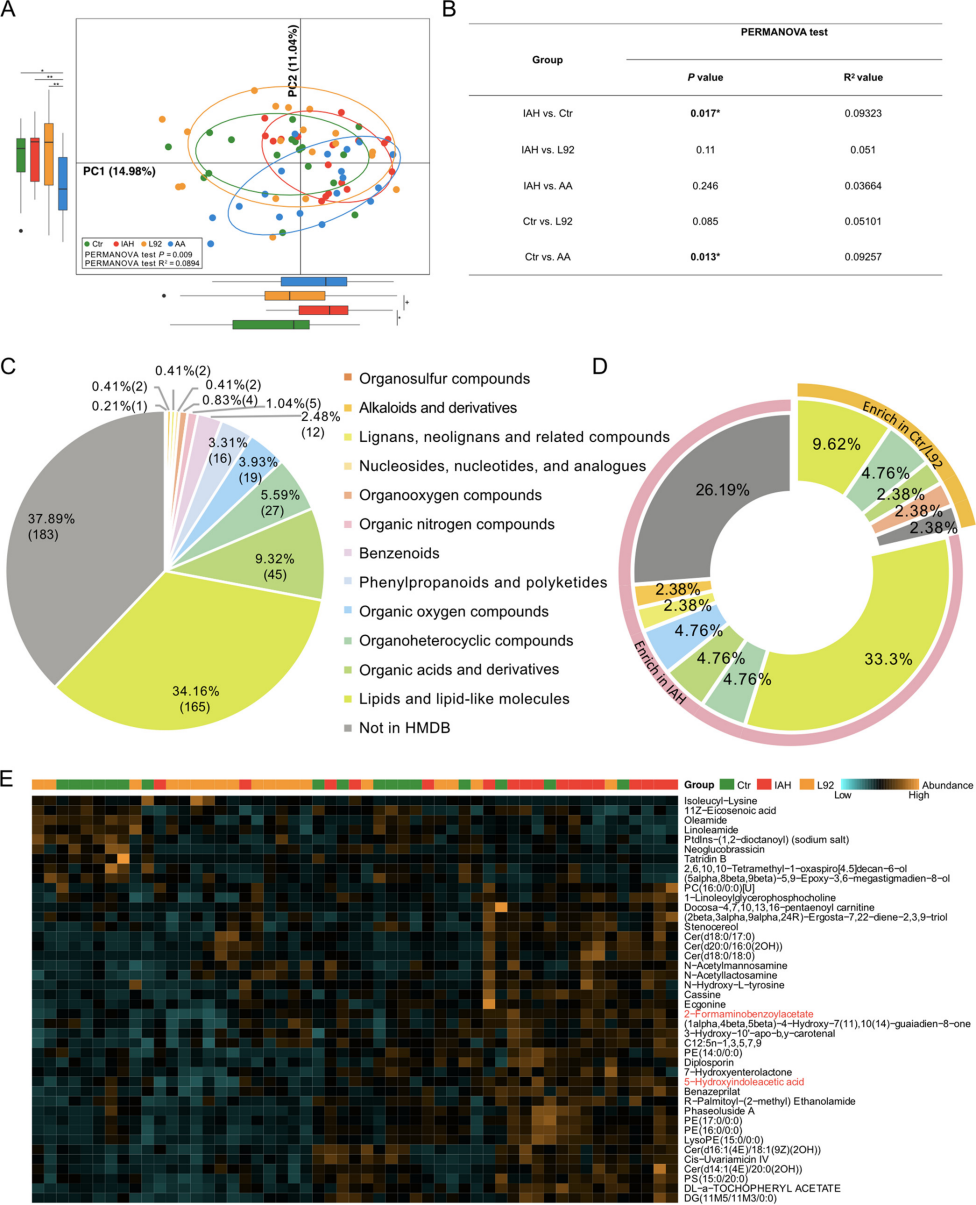
The metabolomic profile of the L92 group was similar to that of the control group. (A) PCA of the IAH, control, L92, and AA groups based on metabolite profiles (*, *P < *0.05; **, *P < *0.01). (B) Analysis of similarity was calculated based on the metabolite abundance profiles. The significance of a comparison of two groups was evaluated using 999 permutations. (C) HMDB compound classifications of the fecal metabolites identified by LC-MS untargeted metabolomics. (D) Doughnut chart showing the types and proportions of differentially abundant metabolites. Color indicates different types of metabolites. (E) Heatmap depicting the differentially abundant metabolites among the IAH, L92, and control groups. Columns are ordered by hierarchical clustering. Each lattice represents the relative abundance of the microbe in a sample; yellow indicates a high abundance, and blue indicates a low abundance. The heatmap is scaled by row. PC, Phosphocholine; PE, Phosphatidylethanolamine; PS, Phosphatidylserine; DG, Diacylglycerol.

We next investigated the fecal metabolome to further elucidate the potentially protective mechanisms of L92 and AA pretreatments. We performed nontargeted metabolomic profiling of stool samples from the four groups (see Materials and Methods for details). A total of 439 metabolites were annotated (Table S1), including 300 metabolites assigned to putative molecular classes based on comparisons with the Human Metabolome Database (HMDB) (Fig. 4A to C). PCA revealed significant differences between the control and IAH groups. Although neither L92 nor AA pretreatment significantly prevented IAH-associated metabolome changes, the metabolomic profile of the L92 group was similar to that of the control group, while that of the AA group was not.

10.1128/msystems.01204-21.9 TABLE S1 Metabolome data set. Download Table S1, XLS file, 0.7 MB. Copyright © 2022 Li et al. 2022 Li et al. https://creativecommons.org/licenses/by/4.0/ This content is distributed under the terms of the Creative Commons Attribution 4.0 International license.

**FIG 5:**
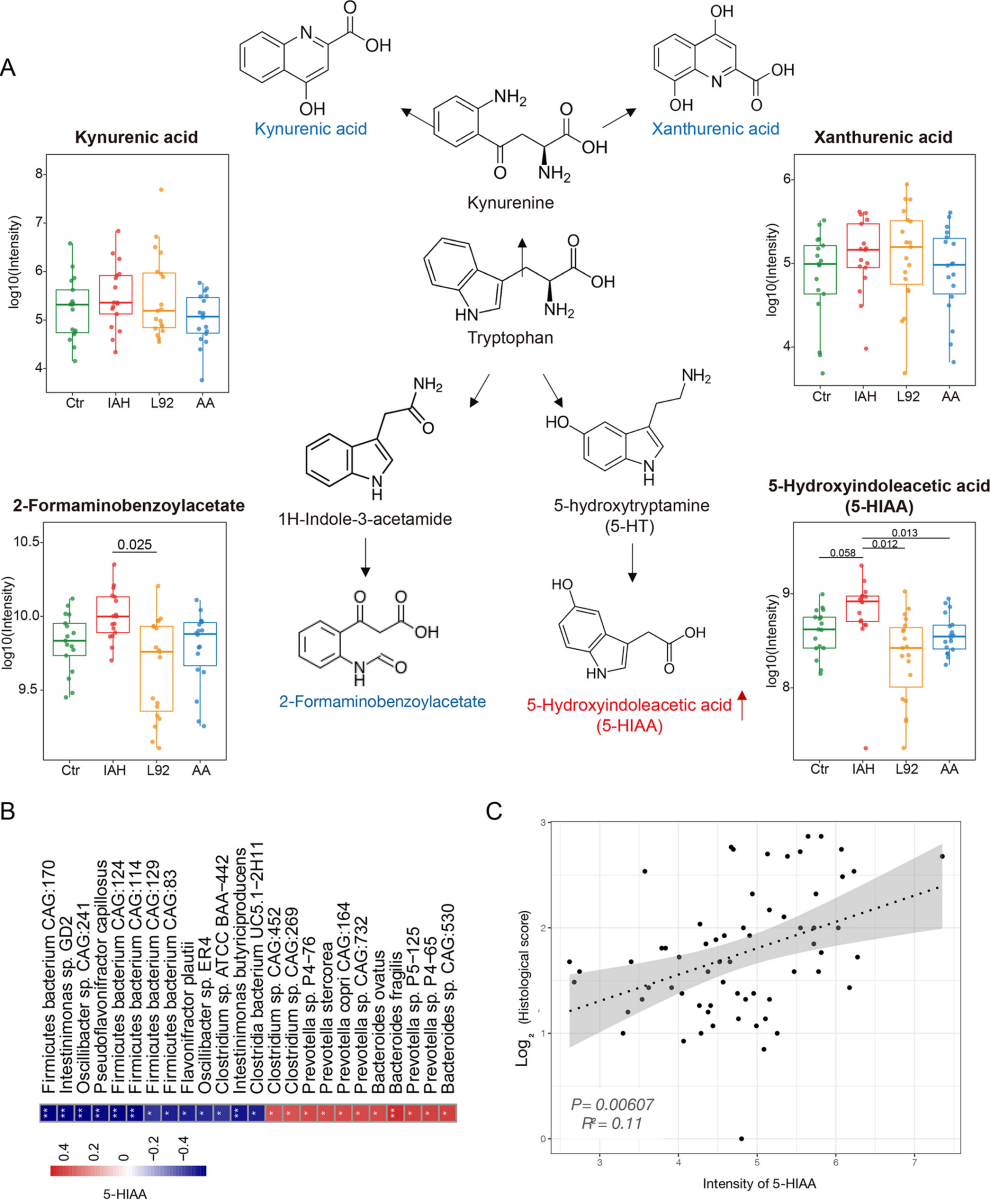
The Trp metabolite 5-HIAA is associated with microbiota disturbances and intestinal histological damage. (A) Simplified tryptophan metabolism pathway labeled with the profiled metabolites. Box plots show the abundances of kynurenic acid, xanthurenic acid, 2-formaminobenzoylacetate, and 5-HIAA in the four groups. (B) Spearman’s correlation analysis between 5-HIAA and the gut microbes. Positive and negative correlations are shown in red and blue in the heatmap, respectively (*, *P < *0.05; **, *P < *0.01 [all *P* values were adjusted by FDR correction]). (C) Correlation analyses between 5-HIAA and histological damage.

Thirty-three metabolites were significantly elevated in the IAH group; 9 metabolites were significantly elevated in both the L92 and control groups (Fig. 4D and E). Interestingly, 2 of the 3 commonly altered metabolites in the L92 and AA groups, 5-hydroxyindoleacetic acid (5-HIAA) and 2-formaminobenzoylacetate (the aromatic hydrocarbon receptor in Trp pathways), are involved in the Trp metabolism pathway (Fig. S5E to G). We therefore examined the patterns of all profiled Trp-related metabolites (kynurenic acid, xanthurenic acid, 2-formaminobenzoylacetate, and 5-HIAA). 5-HIAA and 2-formaminobenzoylacetate showed similar patterns among the different groups, and 5-HIAA was the most promising because IAH-induced increases in 5-HIAA can be prevented by both L92 and AA with statistical significance when compared among all four groups (Fig. 5A). Targeted metabolomic analysis further verified the importance of 5-HIAA (Fig. S6).

10.1128/msystems.01204-21.6 FIG S6 Information on targeted metabolomic analysis. (A) Standard curve of 5-HIAA. (B) Extracted ion current (XIC) of 5-HIAA. (C) Box plots showing the intensities of 5-HIAA measured by the targeted method in four groups. The *P* values were calculated by the Wilcoxon rank sum test. (D) Plots of the linear regression between the 5-HIAA intensities measured by the untargeted and targeted methods. The gray area represents the standard error. Download FIG S6, TIF file, 0.4 MB. Copyright © 2022 Li et al. 2022 Li et al. https://creativecommons.org/licenses/by/4.0/ This content is distributed under the terms of the Creative Commons Attribution 4.0 International license.

### 5-HIAA is associated with microbiota changes.

To assess the overall associations between the microbiota and the metabolome, we first performed Procrustes analysis. There was a consistent and significant inter-omics relationship between the metabolome and the microbiota across the different groups (Fig. S7A) (*M*^2^ = 0.82; *P < *0.001). Next, we investigated the correlations between differentially abundant species and metabolites (Fig. S7B). Differentially abundant species with relative abundances of >0.0005 were selected. A total of 780 false discovery rate (FDR)-significant (adjusted *P < *0.05) associations between representative differentially abundant species and metabolites were observed. We specifically focused on the associations between 5-HIAA and differentially abundant species (Fig. 5B). Twenty-four differentially abundant species showed strong associations (rho value greater than 0.4 or less than −0.4) with 5-HIAA. Specifically, several species of *Prevotella*, B. fragilis, and Bacteroides ovatus enriched in IAH rats were also positively correlated with 5-HIAA, while Flavonifractor plautii and Intestinimonas butyriciproducens were negatively correlated (Fig. S7C). These results further showed that Trp-related metabolites, especially 5-HIAA, are associated with IAH-induced microbiota changes and the protective effects of L92 and AA pretreatments (Fig. 5C).

10.1128/msystems.01204-21.7 FIG S7 Integrated analysis of the gut microbiota and metabolome. (A) Procrustes analysis showing the correlations between the microbiome (open circles) and the metabolome (filled circles). Colors represent different groups. (B) Heatmap showing the correlations between differentially abundant species and metabolites. Positive and negative correlations are shown in red and blue in the heatmap, respectively (*, *P < *0.05; **, *P < *0.01; ***, *P < *0.001 [all *P* values are adjusted by FDR correction]). (C) Examples of different species correlating with the level of 5-HIAA. The colors of points represent grouping information as shown in panel A. Download FIG S7, TIF file, 1.7 MB. Copyright © 2022 Li et al. 2022 Li et al. https://creativecommons.org/licenses/by/4.0/ This content is distributed under the terms of the Creative Commons Attribution 4.0 International license.

### Exploring 5-HIAA as a clinical biomarker for IAH and sepsis.

**FIG 6:**
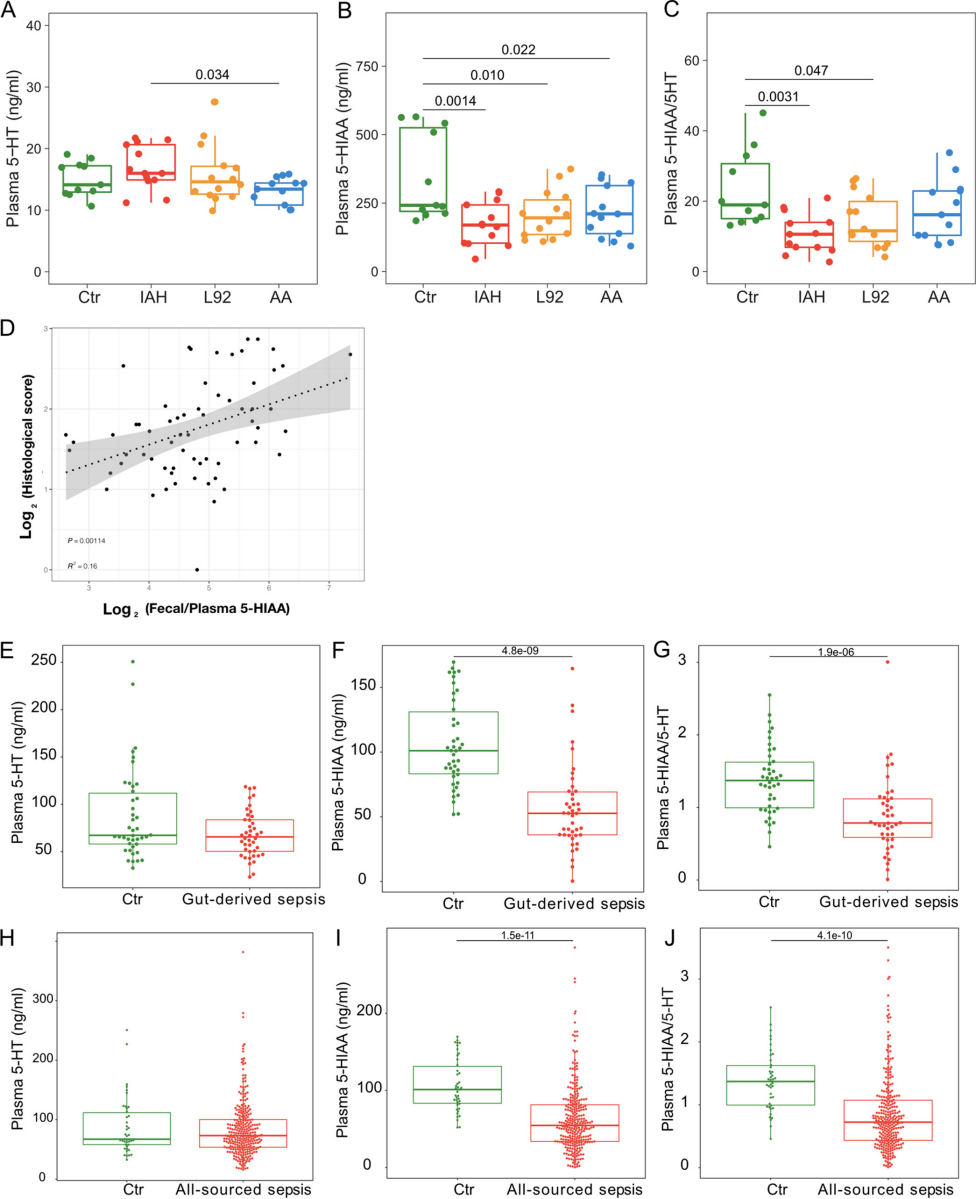
Circulatory changes of 5-HT and 5-HIAA in IAH rats and sepsis patients. (A to C) Concentrations of plasma 5-HT (A) and 5-HIAA (B) and 5-HIAA/5-HT ratios (C) in the Ctr, IAH, L92, and AA groups. (D) Correlation analysis of the ratio of fecal to plasma 5-HIAA and histological damage. (E to G) Concentrations of plasma 5-HT (E) and 5-HIAA (F) and 5-HT/5-HIAA ratios (G) in gut-derived sepsis patients (*n* = 41) compared to the control group (*n* = 42). (H to J) Concentrations of plasma 5-HT (H) and 5-HIAA (I) and 5-HT/5-HIAA ratios (J) in all-source sepsis patients (*n* = 293) compared to the control group (*n* = 42).

**FIG 7:**
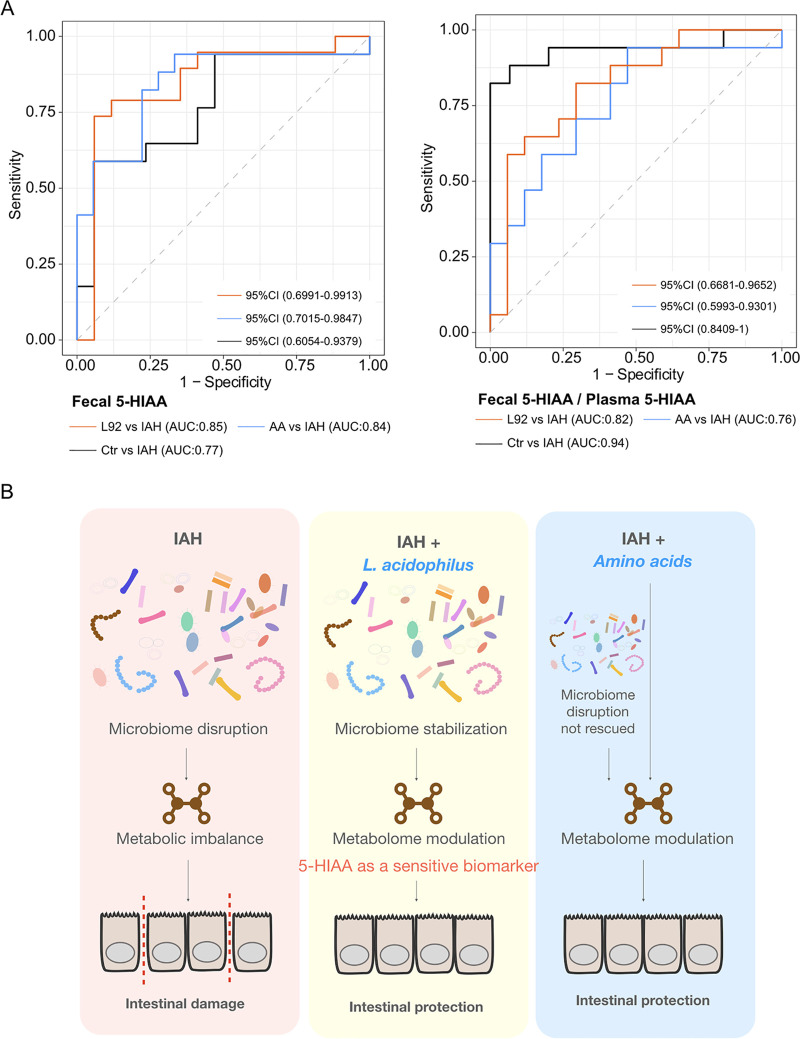
5-HIAA as a potential diagnostic marker and working model of L92- and AA-mediated protective effects. (A) Fecal 5-HIAA and ratio of fecal to plasma 5-HIAA as potential biomarkers for IAH. CI, confidence interval; AUC, area under the curve. (B) Working model of L92- and AA-mediated protective effects demonstrating that microbiome and metabolic modulation can prevent IAH-induced intestinal damage with 5-HIAA as a sensitive biomarker.

To investigate if 5-HIAA and related metabolites can be viable clinical diagnostic biomarkers for IAH, we profiled plasma 5-HIAA and 5-hydroxytryptamine (5-HT), a precursor of 5-HIAA, in rats. In contrast to fecal 5-HIAA, the level of plasma 5-HIAA was significantly decreased in the IAH group, resulting in a significantly lower ratio of plasma 5-HIAA to 5-HT in the IAH group (Fig. 6A to C). Intrigued by these results, we explored the suitability of Trp-related metabolites as diagnostic biomarkers for IAH. Fecal 5-HIAA performed well in distinguishing IAH from the L92- or AA-pretreated groups (Fig. 7A). The ratio of fecal 5-HIAA to plasma 5-HIAA produced a significant improvement in the classification accuracy of IAH from the control (Fig. 7A) and correlated better with the histological damage score (Fig. 6D). Clinical IAH often leads to gut-derived sepsis in patients; we therefore profiled the plasma 5-HT and 5-HIAA of a retrospective cohort of sepsis patients (Table 1) (gut-derived sepsis, *n* = 41; all-source sepsis, *n* = 293) with a matched healthy control cohort (*n* = 42). Strikingly, sepsis patients shared the same changes in plasma 5-HT and 5-HIAA with IAH rats. Specifically, marked decreases in plasma 5-HIAA and the 5-HIAA/5-HT ratio (Fig. 6E to J) were observed in both cohorts of patients. These results demonstrated the feasibility of measuring 5-HIAA as a diagnostic marker for IAH and even sepsis.

**TABLE 1:** Demographic characteristics of the clinical patients^*a*^

Feature	Value for group		
	Control (*n* = 42)	All-source sepsis (*n* = 293)	Gut-derived sepsis (*n* = 41)
Mean age (yrs) ± SD	64.88 ± 13.51	70.60 ± 15.50	68.15 ± 19.73
No. (%) of patients of gender			
Male	30 (71.43)	168 (57.34)	24 (58.54)
Female	12 (28.57)	125 (42.66)	17 (41.46)
No. (%) of patients with outcome			
Shock		45 (15.36)	7 (17.07)
MODS		24 (8.19)	0
Mortality		43 (14.68)	2 (4.88)
Mean length of hospital stay (days) ± SD		13.38 ± 10.12	11.27 ± 7.61
Mean SOFA score upon admission ± SD		3.64 ± 3.26	3.68 ± 3.18
Mean APACHE II score upon admission ± SD		13.13 ± 7.96	10.61 ± 7.62

a MODS, multiple-organ disorder syndrome; SOFA, sepsis-related organ failure assessment; APACHE, acute physiology and chronic health evaluation. The mean ages and sex ratios in the control, all-source sepsis, and gut-derived sepsis groups were not significantly different.

## DISCUSSION

IAH frequently develops in critically ill patients, which induces and aggravates dysfunctions of all organ systems, starting with the gut (15), and may eventually lead to sepsis (16–19). Therefore, early prevention of the development of IAH has important clinical significance. In the present study, we showed that both microbiome and metabolomic modulations can prevent IAH-induced intestinal damage and identified 5-HIAA as a key metabolic marker for IAH and gut-derived sepsis.

First, we demonstrated that modification of the microbiome with L92 is an effective approach to mitigate IAH-induced intestinal damage in rats. For example, IAH-enriched B. fragilis, Bacteroides eggerthii, and B. ovatus were previously reported to be involved in inflammatory responses in inflammatory bowel disease (IBD) (20–22). Many species of *Prevotella* and *Clostridium* enriched in IAH were responsible for intestinal/abdominal infections (23–28). On the other hand, L92-enriched Flavonifractor plautii is beneficial in inhibiting the Th2 immune response to alleviate ovalbumin-induced allergy (29). F. prausnitzii was also enriched in the L92 group. Previous studies have shown that *F. prausnitzii* can promote intestinal homeostasis and integrity (30). Among the significantly upregulated KEGG pathways in L92, selenocompounds play key roles in maintaining cellular redox homeostasis (31), and appropriate dosages of arginine and ornithine have been reported to show intestinal protective effects during inflammation and sepsis (32, 33) (Fig. 3C). Associations between intestinal protection and microbial community/functional pathways were also observed (see Fig. S4D and Fig. S5D in the supplemental material).

We found that direct supplementation with AA also prevented IAH-induced intestinal damage, which supports our previous findings that amino acid biosynthetic pathways are involved in L92-mediated protective effects (10) (Fig. 7B). This result is clinically important since direct supplementation with AA can circumvent the risk of infection associated with the use of probiotics and fecal microbiome transplantation in immunocompromised patients (34, 35). The common protective effects between AA and L92 might be associated with the 6 similarly modulated metabolic pathways, such as ABC transporters, the adipocytokine signaling pathway, and the prolactin signaling pathway (Fig. S8A to F). Previous studies revealed that these pathways were involved in lipid homeostasis, reduction of vascular endothelial inflammation, and immune regulation, etc. (36–40).

10.1128/msystems.01204-21.8 FIG S8 The six level 3 KEGG pathways significantly changed in both the AA and L92 intervention groups and the impact of litter/affiliation by randomized stimulation. (A to F) Relative abundances of ABC transporters (A), the adipocytokine signaling pathway (B), ferroptosis (C), the peroxisome (D), the prolactin signaling pathway (E), and zeatin biosynthesis (F). (G and H) Density plots of *P* values by an Adonis test obtained from simulation based on species abundance profiles (G) and metabolome profiles (H). Litter information was simulated at random for each sample for 100 simulation times. The Adonis test was performed using 999 permutations based on Euclidean distance matrices. Group information and simulated litter information were used as covariates. The dotted line indicates a *P* value of 0.05. Download FIG S8, TIF file, 1.4 MB. Copyright © 2022 Li et al. 2022 Li et al. https://creativecommons.org/licenses/by/4.0/ This content is distributed under the terms of the Creative Commons Attribution 4.0 International license.

Finally, we discovered that IAH and sepsis shared similar metabolic markers. The incidence rate of IAH (without primary disease) is seriously underestimated (41), and even mild IAH (<12 mm Hg) can cause intestinal damage (15, 19, 42), providing a pathophysiological basis for subsequent gut-derived sepsis (43–45). Left untreated, IAH can exacerbate the incidence rate of sepsis and related multiorgan dysfunctions. We demonstrated that patients with different kinds of sepsis showed changes in plasma 5-HT and 5-HIAA similar to those in the rat IAH models (Fig. 6). These findings were consistent with those in previous reports by Troché et al. (46) and Zeden et al. (47) in that the major tryptophan pathway notably changed in septic shock and the levels of tryptophan metabolites were associated with the progression of sepsis. Considering the heterogeneity of sepsis, plasma and/or fecal 5-HIAA is a promising diagnostic biomarker for sepsis.

## MATERIALS AND METHODS

### Animals.

A total of 80 male specific-pathogen-free (SPF) SD rats (30 random litters from 2,000 simultaneously raised litters, 10 to 12 weeks old, weighing 200 to 250 g), purchased from Vital River Laboratory Animal Technology Co. Ltd. (Beijing, China), were used in this study. All animal experiments were carried out in accordance with the U.S. National Institutes of Health *Guide for the Care and Use of Laboratory Animals* (48). The study was approved by the Peking University Biomedical Ethics Committee, Experimental Animal Ethics Branch (approval no. LA2019013). Before the experiments, the animals were allowed to adapt to the environment for a week. Each rat was kept in a separate cage under standard controlled environmental conditions with 12-h-light/dark cycles and supplied SPF-grade rat chow (Beijing Keao Xieli Feed Co. Ltd., Beijing, China) irradiated by cobalt-60 and autoclaved again to avoid feeding-source microbial intake. Next, the rats were assigned to the following 4 groups (*n* = 20) by randomizing cage numbers and were administered treatment twice daily for 7 consecutive days by intragastric gavage before inducing IAH: (i) control group (Ctr) (sterilized water), (ii) IAH group (sterilized water), (iii) L92-pretreated group (L92 treatment [Lactobacillus acidophilus L-92 at 2.1 × 10^9^ CFU/kg of body weight/day]), and (iv) AA-pretreated group (amino acid treatment [25 g/kg/day]). After 7 days of administration, all rats were deprived of food, but not water, for 12 h before inducing nitrogen pneumoperitoneum for 90 min in the IAH, L92, and AA groups (described below). The control animals were treated with a sham injection. The body weight of rats was recorded on days 0 and 7. Surgical operations were performed under sodium pentobarbital anesthesia (intraperitoneal injection of 40 mg/kg). At harvest, the rats were sacrificed with an overdose of sodium pentobarbital (intraperitoneal injection of 160 mg/kg) to minimize pain.

### Preparation of L92 and AA reagents.

L92, containing the single species L. acidophilus, was purchased from Calpis Co. Ltd. (Tokyo, Japan). According to the online product instructions (https://www.calpis-shop.jp/products/l92_tab.html/) and the methods described previously by Nair and Jacob (49), the rat dose equivalent to the human dose was calculated, and the final dose of L92 used was 2.1 × 10^9^ CFU/kg/day, calculated as 200 × 10^8^ CFU (human-used clinical dose)/60 kg × 6.3/day (human-to-rat conversion coefficient). The AA treatment (trade name Elental) is a type of amino acid mixture and was purchased from Ajinomoto Co. Ltd. (Tokyo, Japan). Clinically, the dosage used was progressively increased from 1/6 to a standard dosage (480 g/60-kg adult/day) for 4 to 10 days. The dose of AA in rats adopted here was the uniform dose, which is half of the equivalent human standard dose. The conversion formula was 480 g/60 kg/2 × 6.3/day. Thus, the final dose of AA in rats was 25 g/kg/day. In brief, one tablet of L92 and one bottle of AA were dissolved in 72 mL and 100 mL of sterile water, respectively, and 2 mL of the L92 or AA solution was then fed to rats twice a day by intragastric gavage for 7 days.

### Establishment of the acute IAH model.

As mentioned previously (2), rats were subjected to a 90-min nitrogen pneumoperitoneum to establish an acute IAH animal model. Briefly, after anesthetizing rats with pentobarbital sodium (40 mg/kg by intraperitoneal injection), the rats were placed in a supine position in a restraint device on a heated operating table to maintain their body temperature at 37°C. Nitrogen pneumoperitoneum was performed by injecting nitrogen using a disposable intravenous infusion needle connected to a microinfusion pump. The mini-infusion pump was connected to a blood pressure monitor to dynamically monitor the intra-abdominal pressure (IAP). When the target IAP was reached, a low flow of nitrogen (1 mL/h) was used to maintain the required IAP. In this study, the target IAP was 12 mm Hg. Control animals were treated with sham injections.

### Sample collection.

After gradual decompression, a laparotomy was performed through a mid-abdominal incision, and a 10-cm-long colon segment was isolated. The colon contents were immediately immersed in liquid nitrogen for 15 min, collected into sterile cryotubes, and stored at −80°C for further experiments. Due to no feces or little feces in some rats when taking samples, a total of 76 stool samples (*n* = 20 in the control group, *n* = 20 in the IAH group, *n* = 18 in the L92 group, and *n* = 18 in the AA group) were collected. The colon tissue was gently rinsed with cold saline and divided into 8 parts for analyses of histology, inflammatory responses, and oxidative responses using the corresponding enzyme-linked immunosorbent assay (ELISA) kits. Moreover, we used pairs of small forceps to peel off the fascia tissue next to the abdominal aorta. Next, we clamped the distal heart of the abdominal aorta with a hemostatic clip and punctured the blood back into a vacuum tube with a blood collection needle to the proximal heart of the abdominal aorta. Finally, 2 mL of rat abdominal aorta arterial blood was collected. Whole blood was centrifuged at 3,000 rpm for 15 min at 4°C, and the supernatant, or the plasma, was stored at −80°C after aliquoting for further experiments.

### Pathological evaluation by hematoxylin and eosin staining.

In order to assess histological damage, conventional hematoxylin and eosin (H&E) staining was performed, and micrographs were obtained to assess the degrees of inflammation infiltration, mucosal edema, and epithelial integrity damage (10). Scoring was performed by three pathologists who were unaware of the experiment. In each slide, five different fields of view (zoomed in at a ×200 magnification) were selected and evaluated. The average score of each slide was recorded as the final score for each sample.

### ELISA.

Plasma diamine oxidase (DAO) and lactic acid (LA) were selected to assess the alteration of intestinal permeability (50, 51). Colonic reduced glutathione (GSH) and malondialdehyde (MDA) were used to evaluate the changes in oxidative responses. Colonic interleukin-1β (IL-1β) was measured to assess the inflammatory responses. 5-HT and 5-HIAA in plasma of rats and patients were detected according to the instructions provided by the manufacturer (Shanghai Bluegene Biotech Co. Ltd., Shanghai, China). In brief, frozen samples were thawed on ice and homogenized in saline (1:10, vol/vol). Next, the homogenates were centrifuged at 3,000 rpm for 10 min at 4°C. The supernatant was collected, and the following steps were performed according to the manufacturer’s instructions. Measurements were performed using a spectrophotometer (Thermo Fisher Scientific, Waltham, MA). Detailed information on the enzyme-linked immunosorbent assay (ELISA) kits is provided in Table S2 in the supplemental material.

10.1128/msystems.01204-21.10 TABLE S2 Detailed information on ELISA kits used in the study. Download Table S2, PDF file, 0.06 MB. Copyright © 2022 Li et al. 2022 Li et al. https://creativecommons.org/licenses/by/4.0/ This content is distributed under the terms of the Creative Commons Attribution 4.0 International license.

### Metagenomics profiling.

All rat stool samples were frozen at −80°C before DNA extraction and analysis. Microbial DNA was isolated for metagenomic analysis using an E.Z.N.A. soil DNA kit (Omega Bio-Tek, Norcross, GA, USA) in a clean fume hood. The final concentration and purity of the extracted DNA were measured by using a NanoDrop2000 spectrophotometer (Thermo Scientific, Wilmington, DE, USA), and the quality of the DNA was checked by 1% agarose gel electrophoresis. The DNA was then fragmented to an average size of about 400 bp using a Covaris M220 instrument (Gene Company Ltd., China) for paired-end library construction. A paired-end library was constructed using the TruSeq DNA sample preparation kit (Illumina, San Diego, CA, USA). The adaptors containing full complements of the sequencing primer hybridization site were ligated to the blunt-end fragments. Paired-end sequencing was performed on the Illumina NovaSeq platform (Illumina Inc., San Diego, CA, USA) at Majorbio Bio-Pharm Technology Co. Ltd. (Shanghai, China) using NovaSeq reagent kits according to the manufacturer’s instructions (www.illumina.com).

### Raw data processing and functional annotation.

Paired-end reads were processed using the MajorBio Co. Ltd. pipeline. Briefly, the paired-end Illumina reads were trimmed of adaptors, and low-quality reads (length of <50 bp, quality value of <20, or having N bases) were discarded by fastp (version 0.20.0 [https://github.com/OpenGene/fastp]). To remove host contamination, reads were mapped to the rat genome (rat_ncbi_Rattus_norvegicus.Rnor_6.0.dna.chromosome) using BWA (http://bio-bwa.sourceforge.net) (52). MEGAHIT (53) (version 1.1.2 [https://github.com/voutcn/megahit]) was used to assemble metagenomic data. Contigs of ≥300 bp were selected for further gene prediction and annotation. The prediction of gene, taxonomy, and functional annotation of each assembled contig was performed using MetaGene (54) (http://metagene.nig.ac.jp/metagene/metagene.html). The predicted open reading frames (ORFs) that were ≥100 bp were retrieved and translated into amino acid sequences using the standard NCBI translation table (http://www.ncbi.nlm.nih.gov/Taxonomy/taxonomyhome.html/index.cgi?chapter=tgencodes#SG1). CD-HIT (55) (http://www.bioinformatics.org/cd-hit/) was used to cluster all predicted genes with 95% sequence identity (90% coverage). The reads that passed quality control were mapped to the representative sequences with 95% identity using SOAPaligner (56) (https://anaconda.org/bioconda/soapaligner), and the gene abundance in each sample was assessed. KEGG annotation was conducted using Diamond (version 0.8.35 [https://github.com/bbuchfink/diamond]) against the Kyoto Encyclopedia of Genes and Genomes database (http://www.genome.jp/kegg/) with an E value cutoff of 1e^−5^. Taxonomic annotation was conducted using Diamond against the NR (nonredundant) database with the same parameters.

### Untargeted metabolomics profiling. (i) Sample preparation.

All samples were thawed at 4°C. Next, 400 μL of a cold methanol solution (methanol-water [3:1, vol/vol]) was added to each 50-mg fecal sample, and the sample was ground by a high-throughput tissue grinder at low temperatures. After vortex mixing, the solution was extracted on ice for 10 min three times and stored at −20°C for 30 min. The sample was then centrifuged for 15 min at 13,000 rpm at 4°C, and the supernatant was transferred to a vial for LC-MS analysis.

### (ii) LC-MS analysis parameters and raw data processing.

The platform for LC-MS analysis was the AB Sciex ultra-high performance liquid chromatographytriple quadrupole-time of flight tandem mass spectrometry system (UPLC-Triple-TOF MS/MS) conducted by MajorBio Co. Ltd. (Shanghai, China). LC conditions were as follows: Ethylene Bridged Hybrid (BEH) C_18_ column (100-mm by 2.1-mm internal diameter [ID], 1.7 μm; Waters, Milford, MA, USA), mobile phase A was water (containing 0.1% formic acid), mobile phase B was acetonitrile-isopropanol (1:1) (containing 0.1% formic acid), the flow rate was 0.40 mL/min, the injection volume was 20 μL, and the column temperature was 40°C. MS conditions were as follows: sample mass spectrometer signal acquisition adopted positive and negative ion scanning modes; the electrospray capillary voltage, injection voltage, and collision voltage were 1.0 kV, 40 V, and 6 eV, respectively; the ion source temperature and desolvation temperature were 120°C and 500°C, respectively; the carrier gas flow rate was 900 L/h; the mass spectrometer scanning range was *m/z* 50 to 1,250; and the resolution was 30,000. After a series of preprocessing on the original data, the raw data were identified by the metabolomics processing software Progenesis QI (Waters Corporation, Milford, MA, USA). The MS and tandem mass spectrometry (MS/MS) information was matched with the metabolic database. The main databases are the Human Metabolome Database (HMDB) (http://www.hmdb.ca/), the METLIN Metabolomics Database (https://metlin.scripps.edu/), other public databases, and self-built databases.

### Targeted metabolomics profiling.

We performed a targeted metabolomic analysis of 5-HIAA with assistance from Shanghai Lu-Ming Biotech Co. Ltd. (Shanghai, China). Briefly, for the fecal samples, a 15-mg sample was added to 500 μL of a cold acetonitrile-isopropanol-water solution (3:3:2, vol/vol/vol, containing the internal standard succinic acid-2,2,3,3-d4) and ground with two cold small steel balls (60 Hz for 2 min). For the plasma samples, a 100-μL sample was added to 300 μL of a methanol-acetonitrile solution (2:1, vol/vol, containing 0.1% formic acid, 0.1 mm/L Butylhydroxytoluene (BHT) and succinic acid-2,2,3,3-d4). Next, the samples were dispersed by an ultrasonic lysis method. Two hundred microliters of the supernatant was collected using an organic-phase pinhole filter (0.22 μm) and transferred to brown vials for LC-MS analysis.

Ultraperformance liquid chromatography-electrospray ionization MS/MS (UPLC-ESI-MS/MS) was utilized as the analytical method for the quantitative detection of metabolites. The platform for LC-MS analysis was the AB Sciex UPLC-Qtrap system. LC conditions were as follows: the column was an Acquity UPLC High strength silica (HSS) Pentafluorophenyl (PFP) column (100-mm by 2.1-mm ID, 1.8 μm; Waters, Milford, MA, USA), mobile phase A was water (containing 0.1% formic acid), mobile phase B was methanol (containing 0.1% formic acid), the flow rate was 0.30 mL/min, the injection volume was 5 μL, and the column temperature was 40°C. Gradient elution procedures were 0 min A/B (99:1, vol/vol), 1 min A/B (99:1, vol/vol), 6 min A/B (5:95, vol/vol), 7 min A/B (5:95, vol/vol), 7.01 min A/B (99:1, vol/vol), and 8 min A/B (99:1, vol/vol). MS conditions were as follows: nitrogen was employed as the collision gas, with ion source gas 1 at 50 lb/in^2^, ion source gas 2 at 50 lb/in^2^, and curtain gas (CUR) at 30 lb/in^2^, and the Turbo ion spray source temperature was 450°C.

Target metabolites were analyzed in multiple-reaction monitoring (MRM) mode. The MRM parameters were optimized for each analyte. Data acquisition and further analyses were conducted using Sciex OS-MQ software (AB Sciex, USA).

### Statistical analysis.

To assess the potential impact of affiliation on the results, we simulated randomized litter information 100 times for each rat and performed an Adonis test on the taxonomy data and metabolome data using both group information and simulated litter information as covariates. The results show that while the group information is almost always significant, the litter information hardly makes a difference (see Fig. S8G and H in the supplemental material). For two-group comparisons, unpaired Student’s *t* test was applied using GraphPad Prism (version 6.0). For multigroup comparisons, ordinary one-way analysis of variance (ANOVA) with a *post hoc* Tukey HSD test was performed to evaluate the differences in means between different groups. Data with error bars are represented as means ± standard errors of the means (SEM). An adjusted *P* value of <0.05 was considered statistically significant. To compare the intragroup variations, Bray-Curtis dissimilarity was performed via vegdist in the R package vegan (2.5-7). For dimension reduction, principal-component analysis (PCA) was applied using the R package ade4 (1.7-16). Permutational multivariate analysis of variance (PERMANOVA) in the Adonis function in the R package ade4 (1.7-16) was used to determine significance in dissimilarity matrices across samples. Procrustes analysis using the Procrustes function in the R package vegan (2.5-6) was used to determine inter-omics (microbiome and metabolome) correlations. Spearman correlation analysis was performed on significantly different metabolites and species to assess their correlations by the R package stats (4.1.0). The *P* values of the Spearman analysis were adjusted using the FDR correction by the R package fdrtool (1.2.15). Tukey’s HSD test was used to identify microbial species, pathways, and enzyme features that were differentially abundant in different groups by the R package stats (4.1.0). An adjusted *P* value of <0.05 is considered statistically significant. For metabolic difference analyses, we carried out a Wilcoxon rank sum test. *P* values were adjusted using the FDR correction by the R package fdrtool (1.2.15). The receiver operating characteristic (ROC) analysis was performed with the R package pROC (1.16.2). GraPhlAn (1.1.3) was used to construct the phylogenetic tree. Cytoscape (3.8.2) was performed to construct the cooccurrence network.

### Human subjects.

To examine the clinical relevance of 5-HIAA and 5-HT with sepsis, all-source sepsis patients (*n* = 293), including patients with gut-derived sepsis (*n* = 41), between January 2013 and March 2020 were recruited. For controls, 42 healthy volunteers were recruited. The samples and clinical information in this study were obtained with the approval of the institutional review board (Ethics Committee of Xin Hua Hospital Affiliated to Shanghai Jiao Tong University School of Medicine, approval no. XHEC-D-2021-005). The inclusion criterion was a diagnosis of sepsis, which was defined by sepsis 3.0 criteria (57). The exclusion criteria included pregnancy, breastfeeding, age of <18 years, human immunodeficiency virus infection, treatment with corticosteroids, chemotherapy within 28 days, expected death within 24 h, and mental illness or treatment with psychotropic drugs or other conditions affecting tryptophan metabolism. The clinical data included sepsis-related organ failure assessment (SOFA) and acute physiology and chronic health evaluation II (APACHE II) scores upon admission and length of hospitalization, etc. The blood samples of patients were collected within the first 24 h after diagnosis.

### Data availability.

The sequencing data were uploaded to the public National Center for Biotechnology Information (NCBI) (http://www.ncbi.nlm.nih.gov/) database under BioProject accession no. PRJNA756796. The metabolic data are provided in Table S1.
