# Supplementary figures and images for: Multi-omic Profiling Reveals that Intra-abdominal-Hypertension-Induced Intestinal Damage Can Be Prevented by Microbiome and Metabolic Modulations with 5-Hydroxyindoleacetic Acid as a Diagnostic Marker
Source: mSystems. 2022 May 16;7(3):e01204-21. doi: 10.1128/msystems.01204-21 (PMC9238425; doi:10.1128/msystems.01204-21)

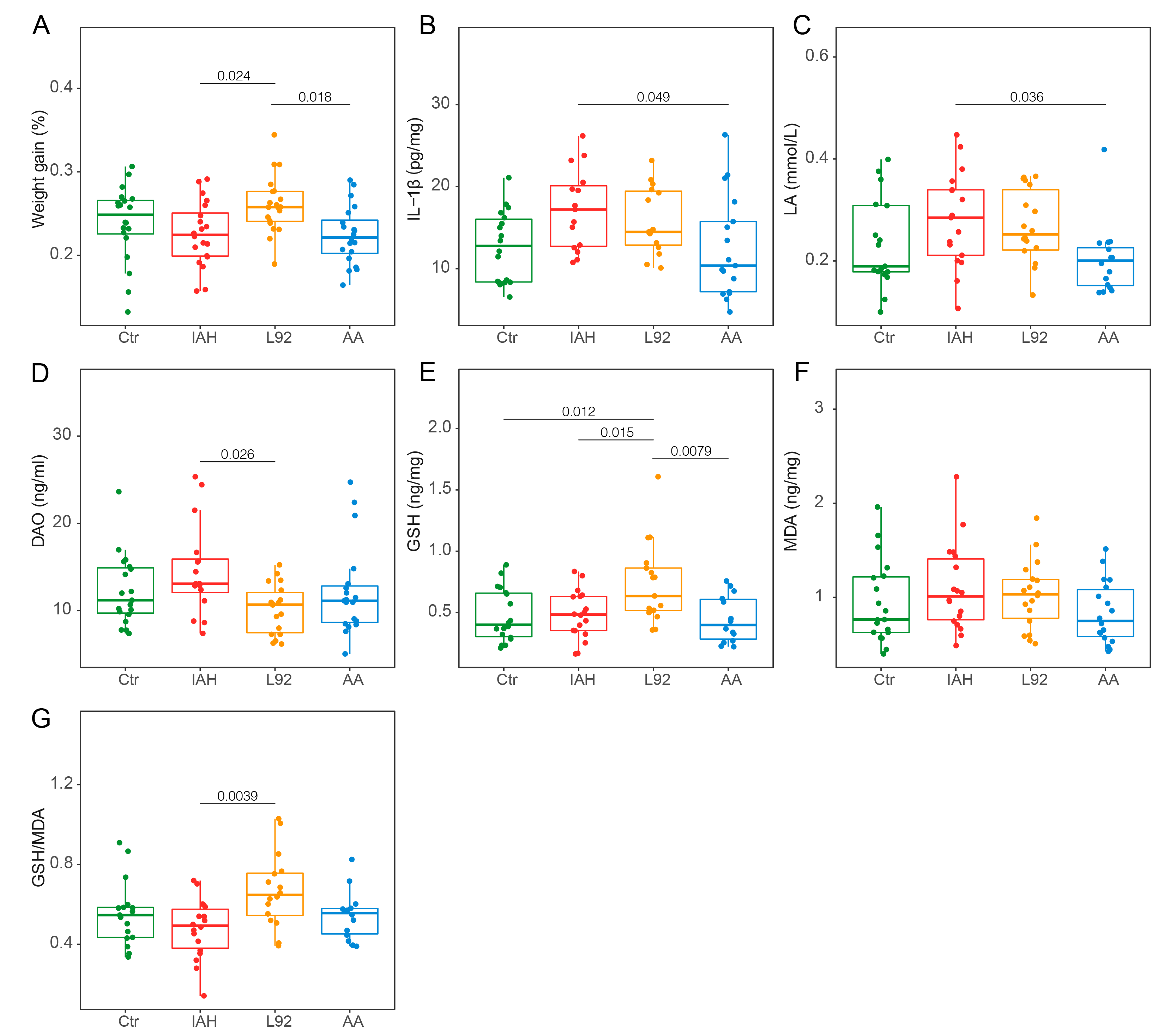

Supplement: FIG S1 [file msystems.01204-21-s0001.tif]

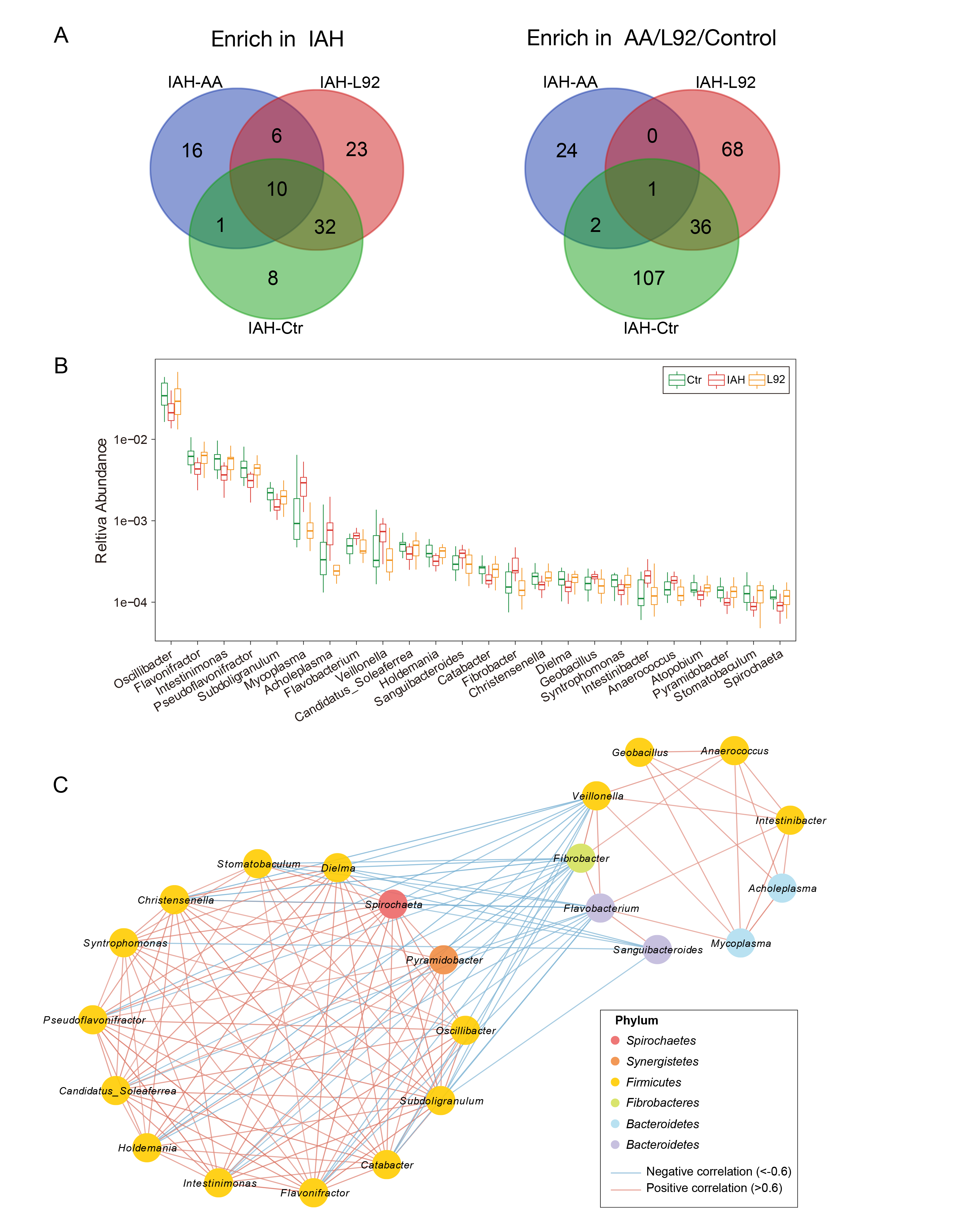

Supplement: FIG S2 [file msystems.01204-21-s0002.tif]

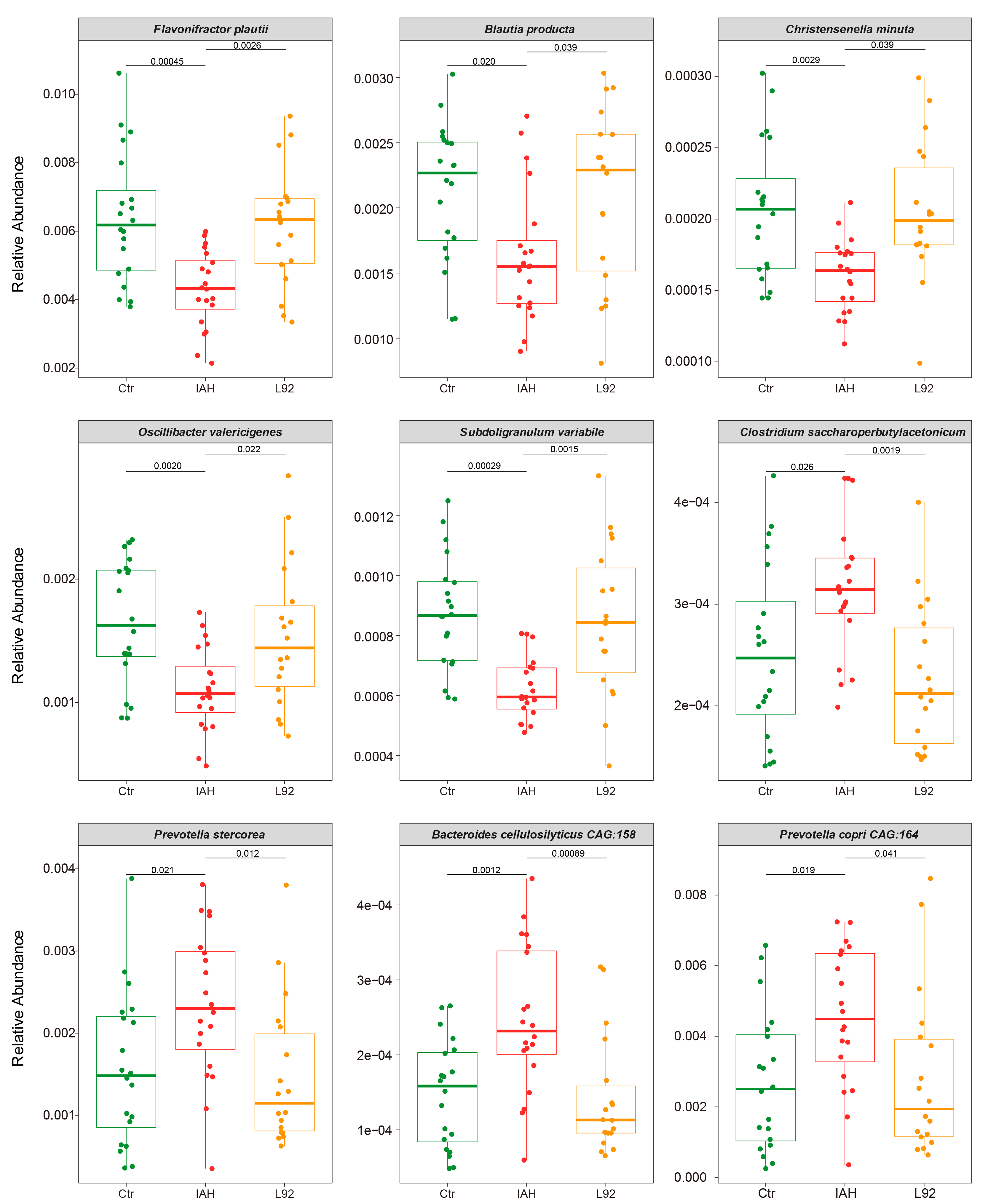

Supplement: FIG S3 [file msystems.01204-21-s0003.tif]

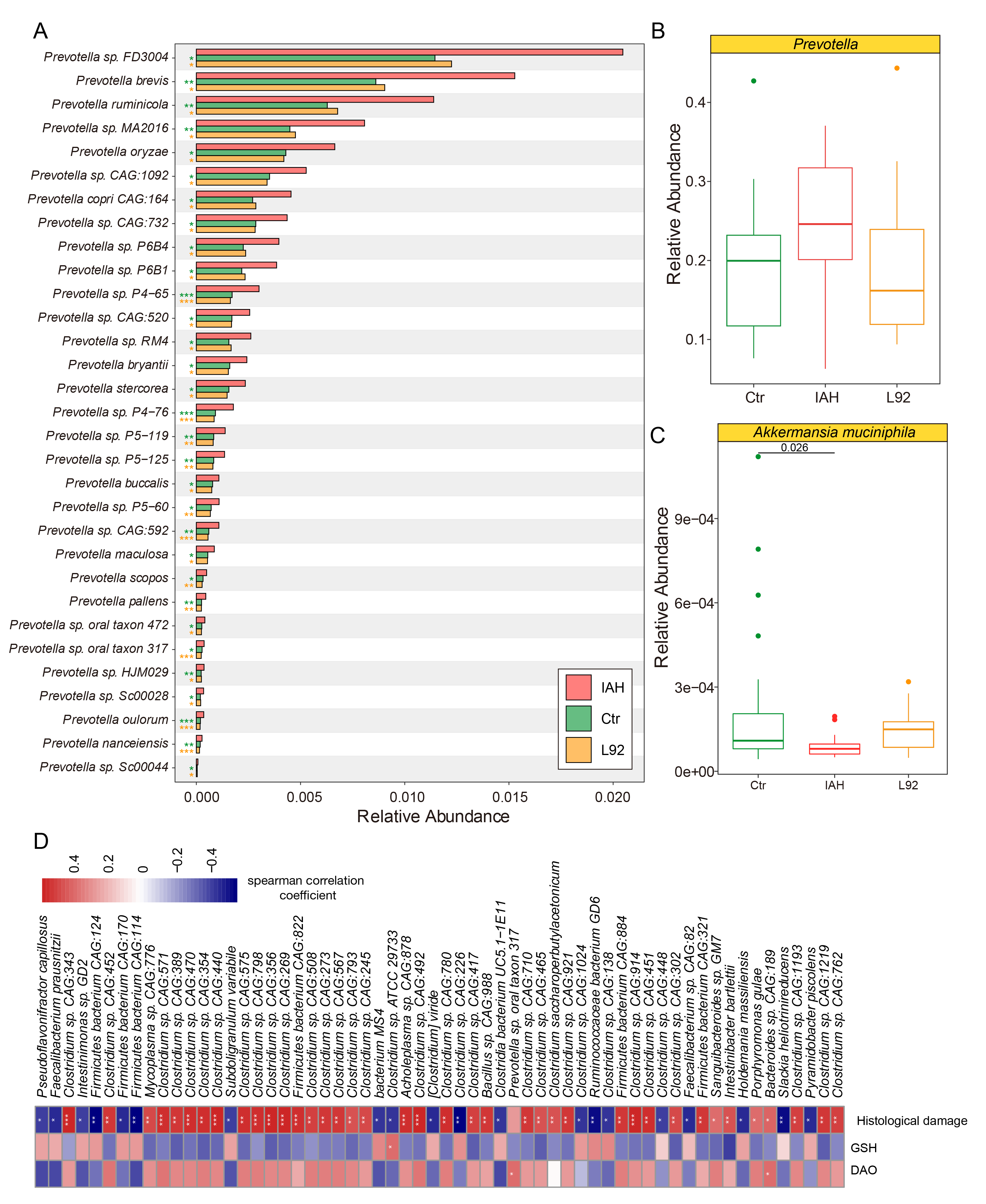

Supplement: FIG S4 [file msystems.01204-21-s0004.tif]

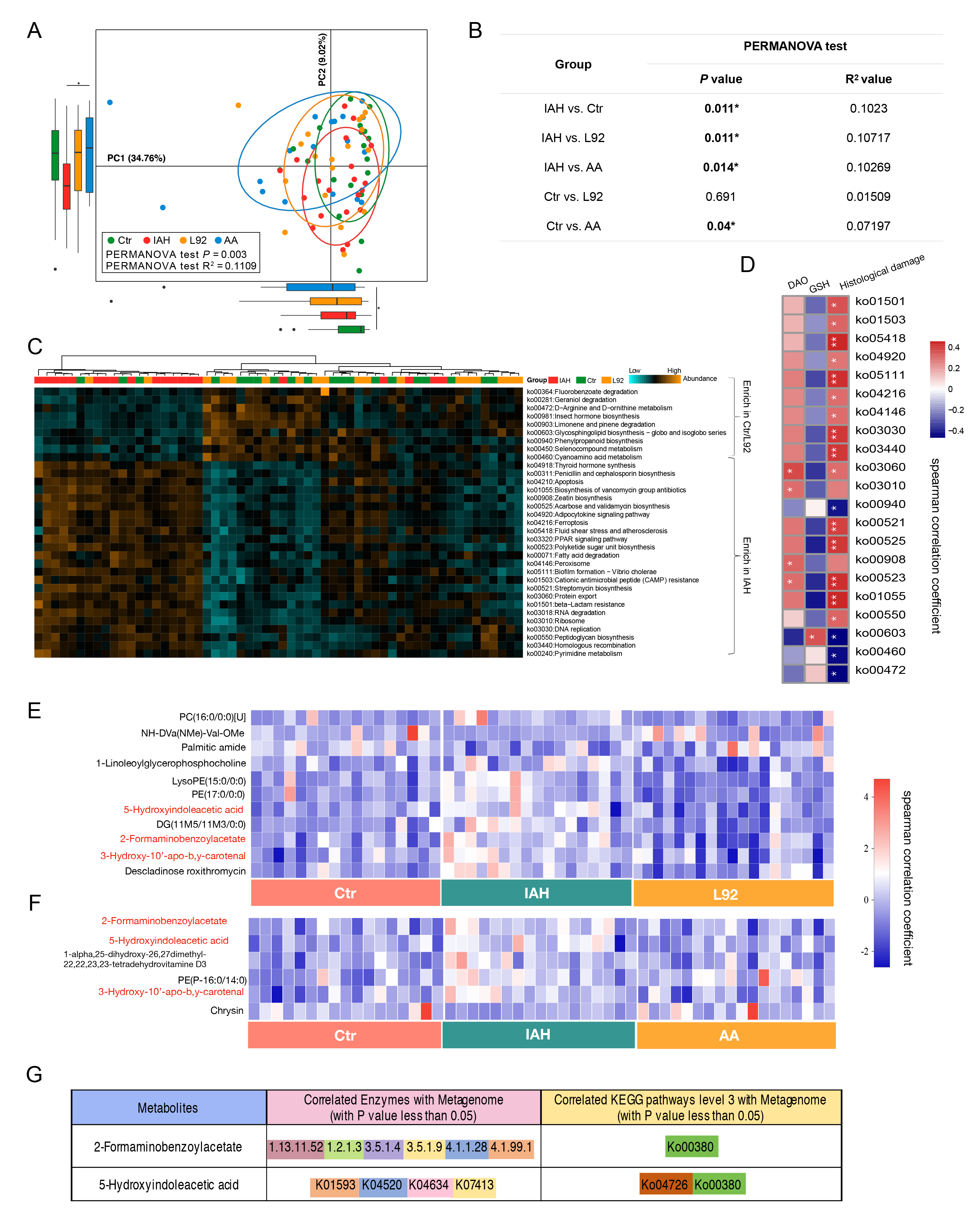

Supplement: FIG S5 [file msystems.01204-21-s0005.tif]

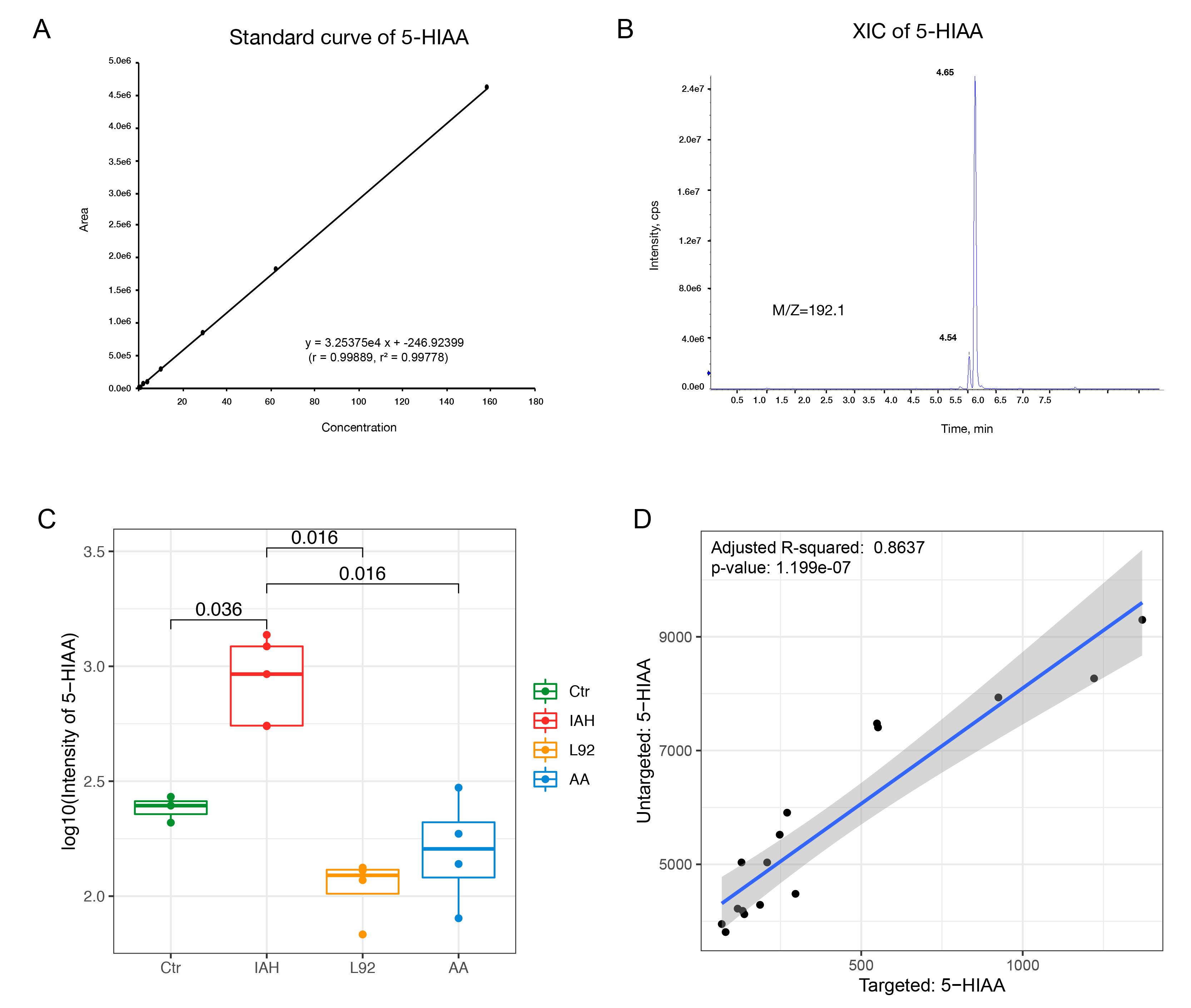

Supplement: FIG S6 [file msystems.01204-21-s0006.tif]

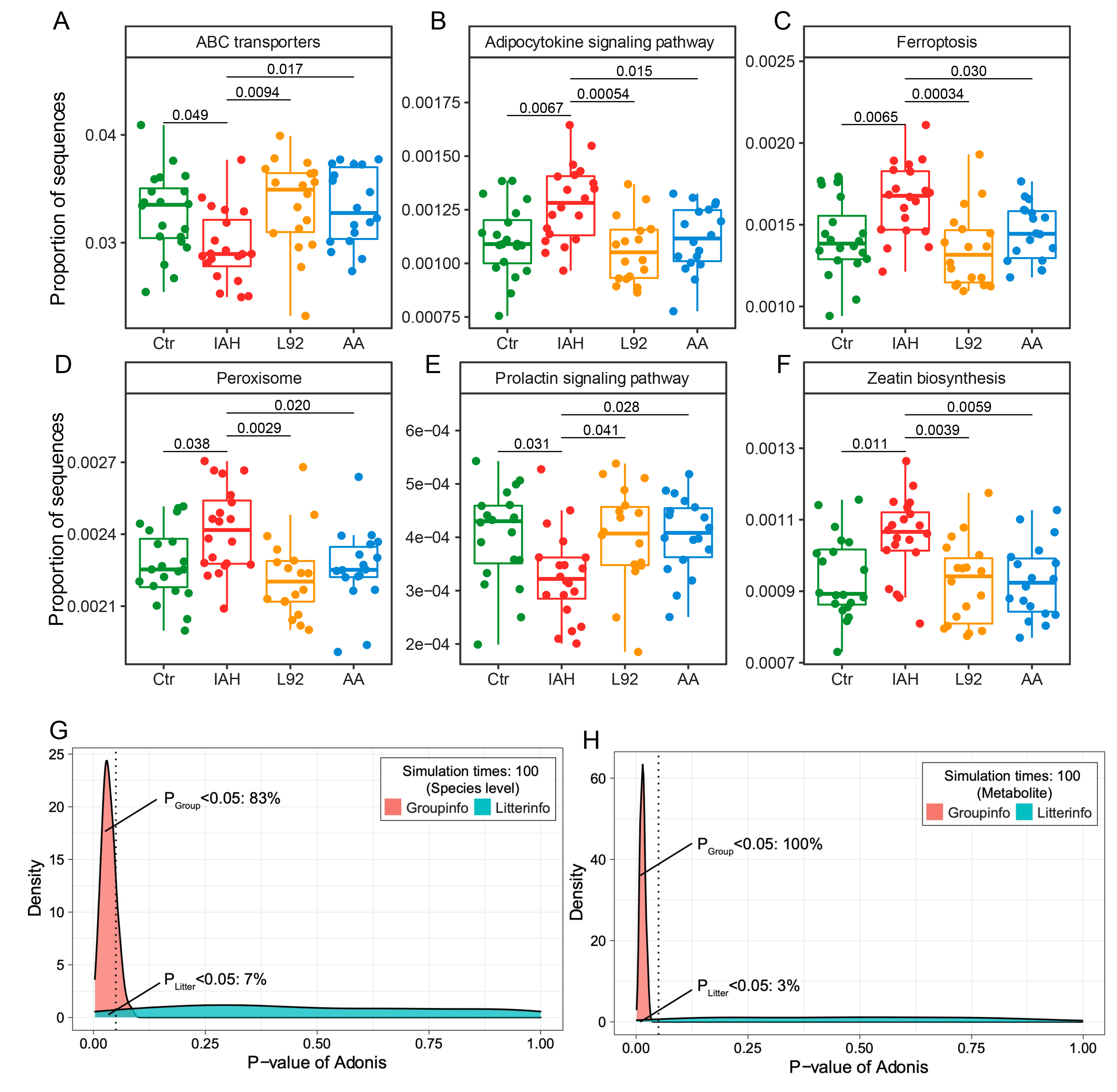

Supplement: FIG S8 [file msystems.01204-21-s0008.tif]
