# Supplementary material for: Multi-omic Profiling Reveals that Intra-abdominal-Hypertension-Induced Intestinal Damage Can Be Prevented by Microbiome and Metabolic Modulations with 5-Hydroxyindoleacetic Acid as a Diagnostic Marker
Source: mSystems. 2022 May 16;7(3):e01204-21. doi: 10.1128/msystems.01204-21 (PMC9238425; doi:10.1128/msystems.01204-21)
Supplement: TABLE S2 [file msystems.01204-21-s0010.pdf]

The detailed information of Elisa kits

Catalog NO.	Product Name	Standard product detection range
E02D0032	Rat Diamine Oxidase (DAO)	0-10 mmol/L
E02L0004	Rat Lactic Acid (LA)	0-1000pg/ml
E02I0010	Rat Interleukin 1β (IL-1β)	0-1000pg/ml
E02G0367	Rat Reduced glutathione (GSH)	0-25ng/mL
E02M0023	Rat Malondialdehyde (MDA)	0-50ng/mL
E02H0106	Rat Hydroxytryptamine (5-HT)	0-100ng/mL
E01H0106	Human 5-Hydroxytryptamine (5-HT)	0-100ng/mL
E02H0320	Rat 5 Hydroxyindoleacetic Acid (5-HIAA)	0-1000ng/mL
E01H0320	Human 5 Hydroxyindoleacetic Acid (5-HIAA)	0-50ng/mL
